# Association of magnesium and vitamin D status with grip strength and fatigue in older adults: a 4-week observational study of geriatric participants undergoing rehabilitation

**DOI:** 10.1007/s40520-023-02450-7

**Published:** 2023-06-07

**Authors:** Eva Kettig, Melanie Kistler-Fischbacher, Caroline de Godoi Rezende Costa Molino, Heike A. Bischoff-Ferrari, Devine Shimbagha Frundi

**Affiliations:** 1 General Internal Medicine, Kantonsspital Winterthur, Winterthur, Switzerland; 2 Department of Aging Medicine and Aging Research, University Hospital Zurich and University of Zurich, Zurich, Switzerland; 3 Center on Aging and Mobility, University Hospital Zurich, Zurich City Hospital Waid and University of Zurich, Zurich, Switzerland; 4 University Clinic for Aging Medicine, Zurich City Hospital Waid, Zurich, Switzerland; 5 Hôpital du Valais, Sion, Switzerland; 6 Berner Institut Für Hausarztmedizin, University of Bern, Bern, Switzerland

**Keywords:** Magnesium, Vitamin D, Grip strength, Fatigue, Rehabilitation, Geriatric

## Abstract

**Background:**

Low magnesium and vitamin D levels negatively affect individuals’ health.

**Aims:**

We aimed to investigate the association of magnesium status with grip strength and fatigue scores, and evaluate whether this association differs by vitamin D status among older participants undergoing geriatric rehabilitation.

**Methods:**

This is a 4-week observational study of participants aged ≥ 65 years undergoing rehabilitation. The outcomes were baseline grip strength and fatigue scores, and 4-week change from baseline in grip strength and fatigue scores. The exposures were baseline magnesium tertiles and achieved magnesium tertiles at week 4. Pre-defined subgroup analyses by vitamin D status (25[OH]D < 50 nmol/l = deficient) were performed.

**Results:**

At baseline, participants (N = 253, mean age 75.7 years, 49.4% women) in the first magnesium tertile had lower mean grip strength compared to participants in the third tertile (25.99 [95% CI 24.28–27.70] vs. 30.1 [95% CI 28.26–31.69] kg). Similar results were observed among vitamin D sufficient participants (25.54 [95% CI 22.65–28.43] kg in the first magnesium tertile vs. 30.91 [27.97–33.86] kg in the third tertile). This association was not significant among vitamin D deficient participants. At week 4, no significant associations were observed between achieved magnesium tertiles and change in grip strength, overall and by vitamin D status. For fatigue, no significant associations were observed.

**Conclusions:**

Among older participants undergoing rehabilitation, magnesium status may be relevant for grip strength, particularly among vitamin D sufficient individuals. Magnesium status was not associated with fatigue, regardless of vitamin D status.

**Study registration:**

Clinicaltrials.gov, NCT03422263; registered February 5, 2018.

**Supplementary Information:**

The online version contains supplementary material available at 10.1007/s40520-023-02450-7.

## Introduction

Magnesium is an essential mineral, which is involved in numerous enzymatic reactions in the body, such as DNA, RNA and protein synthesis, cell growth and reproduction, and cellular energy production and storage [1, 2]. Specifically, magnesium is required for adenosine triphosphate (ATP) synthesis and activation and produces energy for muscle contraction and relaxation. In addition to its critical role for many bodily functions, magnesium is also involved in the regulation, synthesis, and metabolism of vitamin D [3–6]. In fact, low levels of serum magnesium have been associated with vitamin D deficiency [7–10]. Similar to magnesium, vitamin D is involved in the regulation of muscle contraction and energy metabolism [11, 12].

Hypomagnesemia affects approximately 15% of the general population [13] and 25% of hospital inpatients [14], and may contribute to reduced muscle strength. Prevalence of poor muscle strength has been estimated at 48–57% in acute stroke patients [15], 56% in hip fracture patients [16] and 20% in patients with congestive heart failure [17]. Furthermore, prevalence of fatigue in patients with various health conditions requiring rehabilitation, such as heart failure [18], cerebrovascular disease [19], and musculoskeletal conditions [20], is considerably higher compared to the general population.

Given the key role of magnesium and vitamin D in muscle energy metabolism, and the increased prevalence of poor muscle strength, fatigue and low serum magnesium levels in patients with medical conditions requiring rehabilitation, an association between these independent variables cannot be excluded. Indeed, several studies support this association for vitamin D in older adults [12, 21], however, fewer studies have examined serum magnesium levels. One cross-sectional study among 1138 community-dwelling, generally healthy older adults found that baseline magnesium levels were positively associated with hand grip strength, lower extremity strength and muscle power [22]. Fatigue has been described as a symptom of magnesium deficiency [23], however, studies on the association of magnesium status and fatigue among older adults are lacking.

In summary, to the best of knowledge, no study has assessed the association between magnesium levels and muscle strength or fatigue among older rehabilitation patients to date. Furthermore, no studies have examined whether this association is modified by vitamin D status. The aim of the present study was therefore to investigate the association of magnesium status with grip strength and fatigue scores, and to evaluate whether this association differs by vitamin D status among older participants undergoing a 4-week rehabilitation program.

## Methods

### Study design and population

This is a 4-week observational study using data from the PUSH (Performance Under SGLT-2-Inhibitors in Humans, NCT03422263) study. The detailed study protocol has been published [24]. The PUSH study was a 4-week, prospective, observational study among participants with or without type 2 diabetes (T2D), SGLT2i treatment-naïve and with established atherosclerotic cardiovascular disease or at high risk for cardiovascular disease. Furthermore, participants had to be community-dwelling, aged ≥ 40 years and referred to inpatient rehabilitation clinic because of cerebrovascular disease, congestive heart failure or musculoskeletal conditions. Eligible participants were divided into 3 groups: (1) participants with inadequately controlled T2D, who were newly prescribed SGLT2i treatment as part of the study; (2) participants with controlled T2D who did not require new antidiabetic medication; (3) participants without T2D and no treatment. All three study groups underwent a 4-week rehabilitation program (exercise therapy). The study was conducted at the Berner Klinik Montana, Switzerland, between January 2018 and September 2020. For the purpose of the present study, a subsample of PUSH participants aged ≥ 65 years, who were recruited between January 2018 and July 2020, were included and baseline and 4-week follow-up data were used.

The protocol of the PUSH study was approved by the Cantonal Ethical Commission of Bern, Switzerland (BASEC-ID 2017–01724). The research was carried out in accordance with the principles as outlined in the Declaration of Helsinki, and all participants gave written informed consent.

### Clinical assessments

Baseline evaluation was performed within the first two days of rehabilitation admission by clinicians, certified nurses, physiotherapists or occupational therapists. The following data were collected: age, sex, body mass index (BMI), comorbidities, use of walking aids, and main reason for admission. Of these, there were three: ischemic or hemorrhagic stroke, congestive heart failure, or musculoskeletal conditions such as chronic back pain, arthritis including arthroplasty or prosthesis surgery, traumatic injuries (e.g., joint dislocations, fractures, sprains, tendon tears) or repetitive stress injuries (e.g., tendinitis). Comorbidities were assessed using the age-adjusted Charlson comorbidity index (CCI). Scoring of the questionnaire was performed by the same, single investigator at baseline and week 4 according to standard scoring rules. The total CCI score is derived by summing the assigned weights for all comorbid conditions. Higher scores indicate higher level of comorbidities and greater risk of mortality [25].

The use of supplements and medication (e.g., diuretics) was assessed and recorded by the treating physician at baseline and week 4.

### Outcomes

Grip strength of the dominant hand was measured in kilograms, using a manual hydraulic dynamometer (JAMAR Hand Dynamometer®). The average of three consecutive measures was used for analysis [26–28]. Grip strength is a well-validated test [29, 30] and often used to characterize overall limb muscle strength, especially in older adults [31–33].

Subjective fatigue was assessed using the Fatigue Scale for Motor and Cognitive functions (FSMC), a 20-item questionnaire with a total of 100 points developed as a measure of cognitive and motor fatigue [34]. Higher scores indicate a higher level of fatigue. For global fatigue, the total FSMC score and for motor fatigue, the motor fatigue subscale were used. The motor fatigue subscale consists of 10 items and had a maximum score of 50 points [35].

### Blood biomarkers

Fasting venous blood samples were collected from each participant at baseline and at week 4. Serum magnesium, calcium, and creatinine levels were analyzed within 2 h from sample collection at an external laboratory using Roche Diagnostics Cobas 8000 analyzer with a module (c502) for all three markers (Roche Diagnostics, Mannheim, Germany). This is a colorimetric endpoint method whereby the concentration is measured photometrically. The intra- and inter-assay coefficients of variation in the lab were 2.7% and 1.8% for magnesium. The creatinine measures were used to calculate estimated glomerular filtration rate (eGFR) using the 2012 CKD-EPI formula [36, 37].

Serum 25-hydroxy-Vitamin D (25[OH]D) levels were also analyzed within 2 h from sample collection at an external laboratory. A Roche Diagnostics Cobas 8000 analyzer with a module (e801) for 25(OH)D total assay (Roche Diagnostics, Mannheim, Germany) was used, which is a competitive electrochemiluminescence protein binding assay intended for the quantitative determination of total 25(OH)D in human serum and plasma [38]. The intra- and inter-assay coefficients of variation were 2.9% and 5.1% for vitamin D. Following the Endocrine Society clinical practice guideline, vitamin D deficiency was defined as a 25(OH)D measurement of < 50 nmol/l and vitamin D sufficiency as 25(OH)D measurement of ≥ 50 nmol/l [39].

### Statistical analysis

Baseline characteristics are described overall and by tertiles of serum magnesium status at baseline. Categorical variables are presented as frequencies and percentages. Continuous variables are presented as mean and standard deviation (SD). Differences in baseline characteristics between magnesium tertiles were assessed by using χ2 test for categorical variables, and ANOVA for continuous variables.

Separate multivariable linear regression models were used to examine the association of tertiles of magnesium (tertile 1 as the reference group) with each outcome (grip strength, global fatigue scores, and motor fatigue scores) at baseline.

In the longitudinal analyses, separate multivariable linear regression models were used to examine the association of achieved tertiles of magnesium (tertile 1 as the reference group) at week 4 with change from baseline at week 4 for each outcome (grip strength, global fatigue scores, and motor fatigue scores).

All models were adjusted for the treatment group of the original PUSH study (diabetic patients with SGLT2 inhibitor treatment, diabetic patients without SGLT2 inhibitor treatment, non-diabetic patients) [24], sex [40], main reason for admission to rehabilitation (ischemic or hemorrhagic stroke, congestive heart failure or musculoskeletal conditions), and the following baseline covariates: age, BMI, CCI, vitamin D status, and walking aid. For the longitudinal analyses, models were further adjusted for duration of rehabilitation, vitamin D status at week 4, baseline magnesium tertiles, and baseline measure of the outcome.

Pre-defined subgroup analyses by vitamin D status were performed to investigate to what extent vitamin D status would impact the association between tertiles of magnesium status and the three outcomes at baseline and after 4 weeks of follow-up using the same methods and adjustments as described above.

In order to explore the potential effect modification of rehabilitation reason, we tested the interaction between magnesium tertiles and rehabilitation reason for the three different outcomes cross-sectionally and prospectively by adding the respective interaction terms in the models described above. We found no indication of effect modification by rehabilitation reason for any of the exposures and outcomes (Supplemental Table 4). Therefore, no subgroup analysis by rehabilitation reason was performed.

All analyses were carried out using SAS version 9.4 (SAS Institute, Cary, NC). Statistical significance was set at *P* value of < 0.05, and reported *P* values are 2-sided.

## Results

### Population characteristics

A total of 253 study participants with a mean age of 75.7 years (SD 6.3) were included, of which 125 (49.4%) were women (Table 1). Overall, mean BMI was 28.4 kg/m^2^ (5.7), mean CCI was 5.9 (1.4), mean 25(OH)D levels were 48.3 (20.8) nmol/l, mean estimated glomerular filtration rate (eGFR) was 74.9 (24.0) ml/min and mean ionized calcium levels were 1.3 (0.1) mmol/L. More than half of participants had vitamin D deficiency (53.4%) and 59.8% of participants used walking aids. Most participants were referred to rehabilitation due to ischemic or hemorrhagic stroke (37.6%), followed by congestive heart failure (32.8%) and musculoskeletal disorders (29.6%). Ninety (35.7%) participants had diabetes at baseline (defined as HbA1c ≥ 6.5%) and 76 (30.2%) participants took calcium supplements, 169 (66.8%) took vitamin D supplements and 116 (46%) took diuretics at baseline.

**Table 1:** Participants’ characteristics overall and by tertiles of magnesium status at baseline

	Overall (n = 253)	Tertile 1, Mg 0.50–0.79 mmol/L (n = 86)	Tertile 2, Mg 0.80–0.86 mmol/L (n = 85)	Tertile 3, Mg 0.87–1.07 mmol/L (n = 82)	P^a^
Age [years], mean (SD)	75.70 (6.26)	76.63 (6.60)	74.91 (6.09)	75.54 (6.01)	0.19
Sex, n (%)					
Women	125 (49.41)	40 (46.51)	44 (51.76)	41 (50.00)	0.78
Men	128 (50.59)	46 (53.49)	41 (48.24)	41 (50.00)	
BMI overall [kg/m^2^], mean (SD)	28.42 (5.71)	28.24 (5.28)	28.84 (6.08)	28.18 (6.01)	0.71
BMI women [kg/m^2^], mean (SD)	28.03 (5.91)	28.52 (4.83)	28.37 (6.61)	27.20 (6.12)	0.55
BMI men [kg/m^2^], mean (SD)	28.80 (5.50)	28.00 (5.69)	29.35 (5.50)	29.15 (5.30)	0.46
CCI overall, mean (SD)	5.88 (1.43)	6.36 (1.50)	5.71 (1.37)	5.56 (1.29)	< 0.001
CCI women, mean (SD)	5.70 (1.43)	6.25 (1.66)	5.45 (1.27)	5.41 (1.22)	0.01
CCI men, mean (SD)	6.06 (1.41)	6.46 (1.36)	5.98 (1.44)	5.71 (1.35)	0.04
Walking aid, n (%)	150 (59.76)	58 (67.44)	48 (56.47)	44 (53.66)	0.14
Main reason for rehabilitation, n (%)					
Ischemic or hemorrhagic stroke	95 (37.55)	30 (34.88)	36 (42.35)	29 (35.37)	0.10
Congestive heart failure	83 (32.81)	37 (43.02)	22 (25.88)	24 (29.27)	
Muskuloskeletal disorder	75 (29.64)	19 (22.09)	27 (31.76)	29 (35.37)	
25(OH)D levels [nmol/l], mean (SD)	48.26 (20.78)	47.38 (22.14)	49.34 (19.03)	48.14 (21.18)	0.84
Vitamin D deficiency (25[OH]D < 50 nmol/l), n (%)	135 (53.36)	48 (55.81)	41 (48.24)	46 (56.10)	0.76
Fasting plasma glucose [mmol/L], mean (SD)	6.37 (1.37)	6.65 (1.50)	6.39 (1.14)	6.05 (1.39)	0.02
HbA1C [mmol/mol], mean (SD)	45.36 (13.01)	49.96 (16.97)	44.70 (9.33)	41.20 (9.62)	< 0.001

*BMI* body mass index, *SD* standard deviation, *CCI* age-adjusted Charlson comorbidity index, *PUSH* performance under SGLT-2-inhibitors in humans, *SGLT2-inhibitor* sodium dependent glucose co-transporter 2 inhibitor, *25 (OH) D* 25-hydroxyvitamin D. *HbA1C* glycated hemoglobin

^a^Differences between tertiles of magnesium at baseline were assessed by ANOVA test for continuous variables or *X*^2^ test for categorical variables. *P* values are two-sided. Statistical significance is set at *P* < 0.05

At baseline, there were no differences between magnesium tertiles in age, sex, BMI, use of walking aid, main reason for rehabilitation admission, 25(OH)D levels, and vitamin D status (Table 1). There was a statistically significant difference between magnesium tertiles for mean CCI. Participants in the first magnesium tertile had higher mean CCI than participants in the second and third tertiles (6.4, 5.7, and 5.6, respectively, *P* < 0.001).

### Cross-sectional analysis

#### Association of magnesium tertiles with grip strength and fatigue scores at baseline

**Fig. 1:**
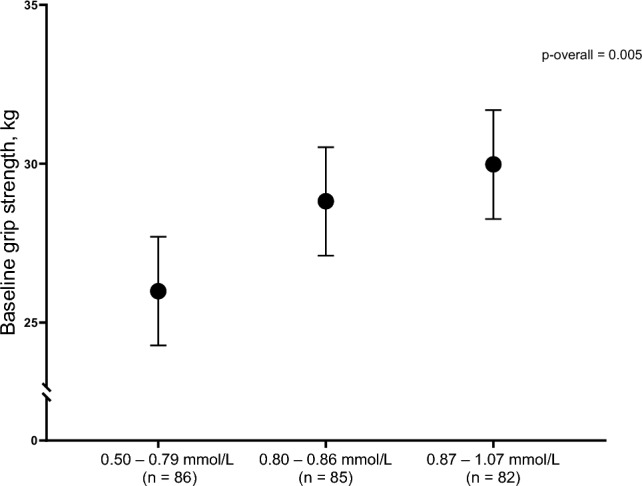
Adjusted^1^ mean grip strength (95% CI) at baseline by tertiles of magnesium. ^1^Adjusted means are least square means (LSM) and 95% CI from multivariable linear regression models. *P* values are from the pairwise comparison between the tertiles with tertile 1 as the reference group. Models were adjusted for treatment group of the original PUSH study, sex, reason for admission to rehabilitation, and baseline covariates: age, BMI, Charlson comorbidity index, vitamin D status (deficiency defined as 25[OH]D < 50 nmol/l), and use of walking aids

At baseline, magnesium tertiles were associated with grip strength (Table 2; Fig. 1), with statistically significant lower grip strength in the first magnesium tertile when compared to the second and third tertiles, even after controlling for relevant confounders (mean grip strength [95% CI]; T1: 25.99 [24.28, 27.70] kg; T2: 28.82 [27.11, 30.52] kg; T3: 29.98 [28.26, 31.69] kg).

**Table 2:** Baseline association of magnesium tertiles with grip strength and fatigue scores^a^

	Tertile 1, Mg 0.50–0.79 mmol/L (n = 86)	Tertile 2, Mg 0.80–0.86 mmol/L (n = 85)	Tertile 3, Mg 0.87–1.07 mmol/L (n = 82)
Grip strength (kg)			
Unadjusted mean (95% CI)	25.98 (23.65, 28.31)	28.56 (26.24, 30.87)	30.10 (27.74, 32.45)
*P*	Ref	0.12	**0.02**
Adjusted mean (95% CI)	25.99 (24.28, 27.70)	28.82 (27.11, 30.52)	29.98 (28.26, 31.69)
*P*	Ref	**0.02**	**0.002**
Fatigue score, global (FSMC)			
Unadjusted mean (95% CI)	52.42 (48.18, 56.66)	58.19 (53.78, 32.59)	55.55 (51.12, 59.99)
*P*	Ref	0.06	0.32
Adjusted mean (95% CI)	52.38 (47.81, 56.95)	56.95 (52.21, 61.69)	56.35 (51.69, 61.01)
*P*	Ref	0.18	0.24
Fatigue score, motor (FSMC)			
Unadjusted mean (95% CI)	27.96 (25.83, 30.09)	31.00 (28.79, 33.21)	28.82 (25.83, 30.09)
* P*	Ref	**0.05**	0.58
Adjusted mean (95% CI)	28.14 (25.85, 30.44)	30.39 (28.02, 32.77)	29.07 (26.76, 31.39)
* P*	Ref	0.19	0.58

Values in bold indicate statistical significance (*p* < 0.05)

*CI* confidence interval, *FSMC* fatigue scale for motor and cognitive functions

^a^Adjusted means are least square means (LSM) and 95% CI from multivariable linear regression models.* P* values are from the pairwise comparison between the tertiles with tertile 1 as the reference group. Models were adjusted for treatment group of the original PUSH study, sex, reason for admission in rehabilitation, and baseline covariates: age, BMI, Charlson comorbidity index, vitamin D status (deficiency defined as 25[OH]D < 50 nmol/l), and use of walking aids

There were no statistically significant associations between magnesium tertiles and global fatigue scores at baseline (Table 2) in the unadjusted and adjusted models. There was, however, a statistically significant association between magnesium tertiles and motor fatigue. On average, participants in the first magnesium tertile had lower fatigue scores compared to the second tertile in the unadjusted model (mean score [95% CI]; T1 27.96 [25.83, 30.09], T2: 31.00 [28.79, 33.21]). After adjusting for confounders, this association was no longer statistically significant (Table 2).

#### Association of magnesium with grip strength and fatigue scores according to vitamin D status at baseline

**Fig. 2:**
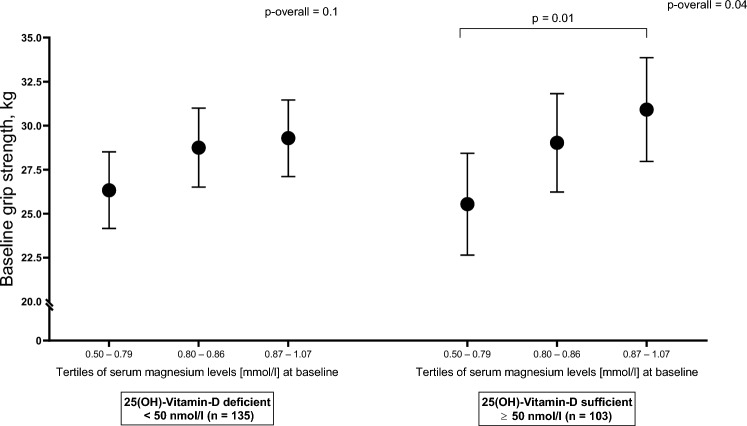
Adjusted^1^ mean grip strength (95% CI) at baseline by tertiles of magnesium according to vitamin D status. ^1^Adjusted means are least square means (LSM) and 95% CI from multivariable linear regression models. *P* values are from the pairwise comparison between the tertiles with tertile 1 as the reference group. Models were adjusted for treatment group of the original PUSH study, sex, reason for admission to rehabilitation, and baseline covariates: age, BMI, Charlson comorbidity index, and use of walking aids

In a subgroup analysis according to participants' vitamin D status, magnesium tertiles were statistically significantly associated with grip strength among participants with vitamin D sufficiency (Fig. 2). On average, mean grip strength was lower in the first magnesium tertile compared to the third tertile (mean grip strength [95%CI]; T1 25.55 [22.65, 28.43] kg; T3: 30.91 [27.97, 33.86] kg), after controlling for potential confounders. Among participants with vitamin D deficiency, no statistically significant association was observed (Online Resource 1, Supplemental Table 5).

There were no statistically significant differences across magnesium tertiles and global or motor fatigue at baseline (Online Resource 1, Supplemental Table 5) when stratified according to vitamin D status.

### Prospective analysis

Out of the 253 participants included at baseline, 238 had serum magnesium level measurements at week 4 and were included in the prospective analyses. Participants were followed for a median time of 29 days. At week 4, mean serum magnesium levels were 0.84 (SD 0.09) mmol/L and mean serum 25(OH)D levels were 53.20 (SD 19.14) nmol/l. Overall mean change from baseline at week 4 in grip strength, fatigue scores, and motor fatigue scores were 0.54 kg, −7.19, −3.99, respectively.

#### Association of achieved tertiles of magnesium levels with change from baseline in grip strength and fatigue

At week 4, there was no statistically significant difference in grip strength change across achieved magnesium tertiles in the unadjusted or adjusted models (Table 3).

**Table 3:** Association of achieved tertiles of magnesium with change from baseline in grip strength and fatigue scores after 4 weeks of rehabilitation^1^

	Achieved tertile 1, Mg 0.50–0.79 mmol/L (n = 78)	Achieved tertile 2, Mg 0.80–0.86 mmol/L, (n = 84)	Achieved tertile 3, Mg 0.87–1.07 mmol/L (n = 76)
Grip strength [kg]			
Unadjusted mean (95% CI) at baseline	27.14 (24.69, 29.59)	28.54 (26.17, 30.91)	28.99 (26.56, 31.42)
*P*	Ref.	0.42	0.29
Unadjusted mean change at week 4 (95% CI)	0.59 (−0.16, 1.34)	0.10 (−0.63, 0.82)	0.76 (0.01, 1.5)
*P*	Ref.	0.35	0.75
Adjusted mean change at week 4 (95% CI)	0.48 (−0.37, 1.33)	0.10 (−0.68, 0.87)	0.75 (−0.11, 1.62)
*P*	Ref.	0.51	0.68
Fatigue score, global (FSMC)			
Unadjusted mean (95% CI) at baseline	51.74 (47.24, 56.24)	57.14 (52.67, 61.60)	57.76 (53.20, 62.32)
*P*	Ref.	0.07	0.09
Unadjusted mean change at week 4 (95% CI)	−4.06 (−7.21, −0.90)	−8.89 (−12.00, −5.78)	−8.87 (−12.06, −5.67)
*P*	Ref.	**0.03**	**0.04**
Adjusted mean change at week 4 (95% CI)	−4.68 (−8.01, −1.36)	−7.55 (−10.50, −4.60)	−7.97 (−11.24, −4.70)
*P*	Ref.	0.21	0.20
Fatigue score, motor (FSMC)			
Unadjusted mean (95% CI) at baseline	27.49 (25.25, 29.73)	30.19 (27.94, 32.45)	30.32 (28.05, 32.59)
*P*	Ref.	0.08	0.10
Unadjusted mean change at week 4 (95% CI)	−2.06 (−3.74, −0.38)	−5.29 (−6.95, −3.63)	−4.65 (−6.34, −2.96)
*P*	Ref.	**0.008**	**0.03**
Adjusted mean change at week 4 (95% CI)	−2.58 (−4.41, −0.76)	−4.43 (−6.04, −2.82)	−4.38 (−6.17, −2.59)
*P*	Ref.	0.14	0.20

Values in bold indicate statistical significance (*p* < 0.05)

*CI* confidence interval, *FSMC* fatigue scale for motor and cognitive functions

^1^Adjusted mean change are least square mean (LSM) and 95% CI from multivariable linear regression models. *P* values are from the pairwise comparison between the tertiles with tertile 1 as the reference group. Models were adjusted for treatment group of the original PUSH study, sex, reason for admission to rehabilitation, duration of rehabilitation, vitamin D status at week 4 (deficiency defined as 25(OH)D < 50 nmol/l), and baseline covariates: age, BMI, Charlson comorbidity index, vitamin D status, use of walking aids, magnesium tertiles, and measure of the outcome

For global fatigue, higher achieved tertiles of magnesium were associated with greater reduction in global fatigue scores at week 4 (mean change [95% CI]; T1: −4.06 [−7.21, −0.90]; T2: −8.89 [−12.00, −5.78]; T3: −8.87 [−12.06, −5.67]). After controlling for potential confounders, this association was no longer statistically significant. Similarly, higher achieved magnesium tertiles were associated with greater reduction in motor fatigue scores at week 4 in the unadjusted model (mean change [95% CI]; T1: −2.06 [−3.74, −0.38]; T2: −5.29 [−6.95, −3.63]; T3: −4.65 [−6.34, −2.96]). After controlling for potential confounders this association was, however, no longer statistically significant (Table 3).

#### Association of achieved tertiles of magnesium levels with change from baseline in grip strength and fatigue according to vitamin D status

After stratifying according to vitamin D status at week 4, no significant associations were found between achieved magnesium tertiles and mean change in grip strength in the unadjusted or adjusted models (Online Resource 1, Supplemental Table 6).

Among participants with vitamin D deficiency, a significantly greater reduction in the global fatigue scores at week 4 was observed in the third achieved magnesium tertile compared to the first achieved magnesium tertile in the unadjusted model (mean change [95% CI]; T1: −2.23 [−7.13, 2.67]; T3: −10.13 [−14.95, −5.31]). No significant association was observed in the adjusted model. Likewise, participants in the higher achieved magnesium tertiles had a greater reduction in motor fatigue scores at week 4 compared to the first achieved magnesium tertile in the unadjusted model (mean change [95% CI]; T1: −0.87 [−3.45, 1.71]; T2: −4.97 [−7.43, −2.51]; T3: −4.93 [−7.52, −2.35]). However, this association was not statistically significant in the adjusted model (Online Resource 1, Supplemental Table 6, Supplemental Fig. 4).

## Discussion

In the present study, we investigated the cross-sectional association of serum magnesium status with grip strength and global and motor fatigue scores and evaluated whether this association differed by vitamin D status among older adults enrolled in the PUSH study, who were undergoing rehabilitation. Furthermore, we examined the prospective association between achieved serum magnesium status and vitamin D status and the changes from baseline in grip strength and fatigue scores after 4 weeks of rehabilitation. Results showed an association between serum magnesium status and grip strength at baseline, with higher tertiles of magnesium being associated with greater grip strength, overall and in the subgroup of vitamin D sufficient participants. Prospective analyses indicated an association between higher achieved serum magnesium tertiles and greater 4-week change in global and motor fatigue, but only in the unadjusted model.

Our findings align with the results from the InCHIANTI study, which reported an association between higher serum magnesium tertiles and greater physical performance, including grip strength, among older adults (i.e., grip strength over 18 months of follow-up [*β* = 2.0 ± 0.5, *P* = 0.0002]) [22]. Participants in the InCHIANTI study (N = 1138) were community-dwelling and slightly younger (mean age 67 years), but otherwise comparable to our study population. Other previous cross-sectional studies explored the associations between dietary magnesium intake, assessed by food frequency questionnaires, and a range of functional performance measurements, including grip strength [41–43]. One of these studies was the Tasmanian Older Adult Cohort Study among community-dwelling adults aged 50–79 years (N = 1099) [41]. Higher magnesium intake at baseline was associated with greater appendicular lean mass (ALM) at baseline and was a positive predictor of changes in ALM over 2.6 years (β = 0.07, *P* = 0.02), however, no associations with knee extensor strength was detected. Similarly, in a cross-sectional study among women aged 18–79 years, highest compared to lowest magnesium intake was associated with greater amounts of fat-free mass (extreme quintile difference: 2.6%, *P* < 0.001; N = 2570), and higher leg extension power (24.1%, *P* < 0.001; N = 1914), but not with grip strength (2.5%, *P* = 0.348; N = 949) [42].

The observation that the association between magnesium status and grip strength persists in the subgroup of vitamin D sufficient individuals but not in those with deficiency, supports a potential interaction effect between the two nutrients [8, 44, 45]. Adequate levels of both, magnesium and vitamin D, may be needed for optimal muscle strength [46–48]. We are not aware of any other, similar, cross-sectional studies which support the interaction effect of magnesium and vitamin D, however, interventional studies support our observation [8]. In a study of 180 individuals (40–85 years old) at risk for colorectal cancer, individualized magnesium supplementation, based on baseline intake, increased the 25(OH)D concentration when baseline 25(OH)D concentrations were 30 ng/mL compared to placebo (mean difference from baseline = 2.79 ng/mL, 95% CI 0.25, 5.34) [8].

To the best of our knowledge, this is the first cross-sectional study which examined the association of serum magnesium status with global or motor fatigue. We detected significant positive associations between mean change in global and motor fatigue score and achieved tertiles of serum magnesium levels in the unadjusted model. A similar, but not statistically significant pattern was observed in the adjusted model, which may suggest that other clinical characteristics are also relevant to fatigue in addition to magnesium status. Cross-sectional studies on the association of serum magnesium levels and fatigue in geriatric patients are lacking, however, evidence from interventional studies in various populations at risk for fatigue support our findings. For example, magnesium supplementation reduced fatigue in women suffering from breast cancer [49] and patients suffering from chronic fatigue syndrome [50]. Furthermore, 6 months of magnesium supplementation improved energy levels compared to placebo, measured by the Nottingham Health Profile Scores, among 32 young (mean age: ~ 36 years) adults (mean difference: −46.57, 95% CI −76.16, −16.98, *P* = 0.002) [51].

When considering vitamin D status, the association between achieved tertiles of magnesium levels and changes in global and motor fatigue score remained significant in vitamin D deficient individuals in the adjusted models. These results are in contrast to the observations for grip strength, where the positive association was only observed in vitamin D sufficient patients, and to previous studies, which have shown a negative association between vitamin D and fatigue in older adults [52, 53]. Further prospective studies with a larger sample size are needed to investigate the association between magnesium status, vitamin D status and fatigue in older adults.

Our findings that magnesium status is associated with grip strength are of clinical relevance since poor muscle strength is a major contributing factor for disability and frailty [54]. Furthermore, the risk of becoming frail increases with the number of micronutrient deficiencies [55]. Screening for magnesium deficiency may therefore be a relevant strategy for successful rehabilitation in geriatric populations at risk of frailty and disability.

The findings on magnesium status and fatigue are less clear and further studies are required. Chronic fatigue presents an important public health problem, and is a prevalent comorbidity of various health conditions such as cancer, rheumatic disorders or, more recently, COVID-19 [56–62]. The assessment of fatigue can be challenging due to its subjectivity, limited standardized assessment tools, and limited knowledge of its pathophysiological pathways. These limitations can contribute to a poor understanding and appreciation of its implications for older people in clinical practice [63]. Future studies on the association of fatigue and nutrient deficiencies may help to guide health care professionals in identifying the most appropriate nutritional recommendations and interventions in the future.

Some limitations of the present study should be acknowledged. First, this was a secondary analysis including older participants from the PUSH study, which was not designed to evaluate the association of magnesium status with grip strength and fatigue. Second, this is an observational study therefore causality cannot be inferred and residual confounding may have biased our results. Third, the follow-up time of four weeks was relatively short and further studies with longer follow-up periods and larger sample sizes are thus needed. Fourth, measuring actual magnesium status and assessing magnesium deficiency is complex, mostly because magnesium is an intracellular cation. Measuring serum concentrations is the clinical standard but often does not accurately reflect magnesium status in the body [64–67]. A validated, accurate and widely available clinical test is lacking. Fifth, generalizability of the study findings may be limited to older patients undergoing rehabilitation. And last, we did not adjust the p-values for multiple testing. Therefore, statistically significant associations may have occurred by chance.

## Conclusions

Among older adults undergoing rehabilitation, higher tertiles of serum magnesium was cross-sectionally associated with greater grip strength overall and in individuals with vitamin D sufficiency, but not among individuals with vitamin D deficiency. The association between magnesium status and global and motor fatigue remains unclear and longitudinal studies are required to examine the role of magnesium alone, and in combination with vitamin D in individuals at risk for fatigue.

## Supplementary Information

Below is the link to the electronic supplementary material.

Supplementary file1 (PDF 182 KB)

## Data Availability

The datasets generated and/or analyzed during the current study are available from the corresponding author on reasonable request.
