# Supplementary material for: Association of magnesium and vitamin D status with grip strength and fatigue in older adults: a 4-week observational study of geriatric participants undergoing rehabilitation
Source: Aging Clin Exp Res. 2023 Jun 7;35(8):1619–29. doi: 10.1007/s40520-023-02450-7 (PMC10245357; doi:10.1007/s40520-023-02450-7)
Supplement: Supplementary file 1 — Supplementary file1 (PDF 182 KB) [file 40520_2023_2450_MOESM1_ESM.pdf]

SUPPLEMENTARY MATERIAL

Supplemental Table 4. P values for the interaction between magnesium tertiles and rehabilitation reason for the three outcomes at baseline and week 4.

Interaction term	Outcome	P value
Baseline Serum magnesium tertiles* rehabilitation reason	Baseline grip strength	0.3742 [1]
	Baseline fatigue	0.9523 [1]
	Baseline motor fatigue	0.9662 [1]
Achieved serum magnesium tertiles * rehabilitation reason	Change in grip strength	0.4721 [2]
	Change in fatigue	0.5263 [2]
	Change in motor fatigue	0.6901 [2]

[1] P-values are from linear regression models for each respective outcome with baseline serum magnesium tertiles and the interaction term between with baseline serum magnesium tertiles and rehabilitation reason as main exposures. Similar to the main analysis, models were adjusted for the treatment group of the original PUSH study (diabetic patients with SGLT2 inhibitor treatment, diabetic patients without SGLT2 inhibitor treatment, non-diabetic patients), sex, main reason for admission to rehabilitation (ischemic or hemorrhagic stroke, congestive heart failure or musculoskeletal conditions) , age, BMI, Charlson comorbidity index, vitamin D status, and walking aid.

[2] P-values are from linear regression models for each respective outcome (change from baseline at week 4) with achieved serum magnesium tertiles and the interaction term between with achieved serum magnesium tertiles and rehabilitation reason as main exposures. Similar to the main analysis, models were adjusted for the treatment group of the original PUSH study (diabetic patients with SGLT2 inhibitor treatment, diabetic patients without SGLT2 inhibitor treatment, non-diabetic patients), sex, main reason for admission to rehabilitation (ischemic or hemorrhagic stroke, congestive heart failure or musculoskeletal conditions), duration of rehabilitation, vitamin D status at week 4 , and the following baseline covariates: age, BMI, CCI, vitamin D status, walking aid, magnesium tertiles, and baseline measure of the outcome.

Supplemental Table 5. Baseline association of magnesium tertiles with grip strength and fatigue scores by vitamin D status (sufficiency 25(OH)D ≥50nmol/L and deficiency 25(OH)D <50nmol/L)[1]

	Tertile 1<br>Mg 0.50 – 0.79 mmol/L	Tertile 2<br>Mg 0.80 – 0.86 mmol/L	Tertile 3<br>Mg 0.87 – 1.07 mmol/L
**Vitamin D deficiency** (n = 135)	n = 48	n = 41	n = 46
**Grip strength [kg]**			
Unadjusted mean (95% CI)	26.07 (22.92, 29.22)	28.40 (25.10, 31.70)	30.09 (26.94, 33.24)
P	Ref.	0.31	0.08
Adjusted mean (95% CI)	26.33 (24.156, 28.51)	28.75 (26.51, 31.00)	29.29 (27.11, 31.46)
P	Ref.	0.14	0.06
**Fatigue Score, global (FSMC)**			
Unadjusted mean (95% CI)	53.96 (47.76, 60.15)	58.74 (52.09, 65.40)	56.41 (49.92, 62.91)
P	Ref.	0.30	0.59
Adjusted mean (95% CI)	53.69 (47.15, 60.23)	58.77 (51.82, 65.73)	56.68 (49.93, 63.42)
P	Ref.	0.31	0.53
**Fatigue Score, motor (FSMC)**			
Unadjusted mean (95% CI)	28.32 (25.28, 31.36)	31.38 (28.16, 34.61)	29.05 (25.90, 32.20)
P	Ref.	0.17	0.74
Adjusted mean (95% CI)	28.38 (25.16, 31.60)	31.43 (28.03, 34.82)	28.92 (25.63, 32.21)
P	Ref.	0.21	0.82

**Vitamin D sufficiency** (n = 103)	n = 34	n = 36	n = 33
**Grip strength [kg]**			
Unadjusted mean (95% CI)	25.98 (22.08, 29.89)	29.13 (25.35, 32.92)	30.00 (26.04, 33.96)
P	Ref.	0.25	0.16
Adjusted mean (95% CI)	25.54 (22.65, 28.43)	29.03 (26.23, 31.82)	30.91 (27.97, 33.86)
P	Ref.	0.09	**0.01**
**Fatigue Score, global (FSMC)**			
Unadjusted mean (95% CI)	50.66 (44.60, 56.71)	54.73 (48.48, 60.98)	55.61 (49.46, 61.76)
P	Ref.	0.35	0.26
Adjusted mean (95% CI)	50.13 (43.70, 56.55)	55.47 (48.96, 61.98)	55.39 (48.85, 61.94)
P	Ref.	0.25	0.27
**Fatigue Score, motor (FSMC)**			
Unadjusted mean (95% CI)	27.78 (24.60, 30.96)	29.24 (25.90, 32.58)	29.29 (26.06, 32.52)
P	Ref.	0.53	0.51
Adjusted mean (95% CI)	27.63 (24.25, 31.01)	29.53 (26.07, 33.00)	29.01 (25.61, 32.41)
P	Ref.	0.44	0.58

Abbreviations: CI, confidence interval. FSMC, Fatigue Scale for Motor and Cognitive Functions.

[1] Adjusted means are least square means (LSM) and 95% CI from multivariable linear regression models. *P* values are from the pairwise comparison between the tertiles with tertile 1 as the reference group. Models were adjusted for treatment group of the original PUSH study, sex, reason for admission to rehabilitation, and baseline covariates: age, BMI, Charlson Comorbidity Index, and use of walking aids.

Supplemental Table 6. Association of achieved tertiles of magnesium with change from baseline in grip strength and fatigue scores by vitamin D status (sufficiency 25[OH]D ≥ 50nmol/l and deficiency 25[OH]D < 50nmol/l) after four weeks of rehabilitation[1]

	Tertile 1 Mg 0.50 – 0.79 mmol/L	Tertile 2 Mg 0.80 – 0.86 mmol/L	Tertile 3 Mg 0.87 – 1.07 mmol/L
**Vitamin D deficiency** (n=106)	n = 32	n = 37	n = 35
**Grip strength [kg]**			
Absolute unadjusted mean (95% CI)	27.44 (22.59, 32.29)	27.78 (24.91, 30.64)	29.77 (25.93, 33.60)
Unadjusted mean change (95% CI)	1.06 (-0.12, 2.24)	-0.23 (-1.32, 0.86)	0.64 (-0.48, 1.77)
P	Ref.	0.11	0.61
Adjusted mean change (95% CI)	1.10 (-0.15, 2.35)	-0.30 (-1.39, 0.79)	0.72 (-0.48, 1.91)
P	Ref.	0.10	0.68
**Fatigue Score, global (FSMC)**			
Absolute unadjusted mean (95% CI)	52.13 (45.38, 58.88)	56.83 (49.72, 63.94)	58.67 (50.32, 67.01)
Unadjusted mean change (95% CI)	-2.23 (-7.13, 2.67)	-7.55 (-12.22, -2.87)	-10.13 (-14.95, -5.31)
P	Ref.	0.12	**0.02**
Adjusted mean change (95% CI)	-3.92 (-8.74, 0.90)	-6.87 (-11.19, -2.56)	-9.44 (-14.17, -4.71)
P	Ref.	0.38	0.13
**Fatigue Score, motor (FSMC)**			
Absolute unadjusted mean (95% CI)	27.33 (23.96, 30.70)	30.26 (26.70, 33.83)	30.06 (26.05, 34.07)
Unadjusted mean change (95% CI)	-0.87 (-3.45, 1.71)	-4.97 (-7.43, -2.51)	-4.93 (-7.52, -2.35)
P	Ref.	**0.02**	**0.03**
Adjusted mean change (95% CI)	-1.83 (-4.39, 0.72)	-4.39 (-6.68, -2.10)	-4.66 (-7.20, -2.11)
P	Ref.	0.15	0.15

**Vitamin D sufficiency** (n = 113)	n = 37	n = 39	n = 36
**Grip strength [kg]**			
Absolute unadjusted mean (95% CI)	28.45 (24.39, 32.51)	29.62 (26.02, 33.22)	28.70 (24.99, 32.42)
Unadjusted mean change (95% CI)	0.03 (-1.04, 1.11)	0.68 (-0.40, 1.75)	0.83 (-0.26, 1.92)
P	Ref.	0.40	0.30
Adjusted mean change(95% CI)	0.06 (-1.16, 1.29)	0.53 (-0.56, 1.62)	0.75 (-0.54, 2.05)
P	Ref.	0.58	0.49
**Fatigue Score, global (FSMC)**			
Absolute unadjusted mean (95% CI)	48.15 (41.37, 54.93)	56.15 (50.30, 61.99)	56.21 (50.05, 62.36)
Unadjusted mean change (95% CI)	-3.29 (-7.89, 1.31)	-8.94 (-13.40, -4.48)	-7.82 (-12.28, -3.36)
P	Ref.	0.08	0.16
Adjusted mean change (95% CI)	-4.80 (-9.87, 0.27)	-8.12 (-12.38, -3.87)	-7.35 (-12.44, -2.25)
P	Ref.	0.32	0.53
**Fatigue Score, motor (FSMC)**			
Absolute unadjusted mean (95% CI)	25.97 (22.57, 29.37)	29.42 (26.28, 32.57)	30.29 (27.42, 33.17)
Unadjusted mean change (95% CI)	-2.43 (-4.93, 0.06)	-4.78 (-7.20, -2.37)	-4.67 (-7.04, -2.29)
P	Ref.	0.18	0.20
Adjusted mean change (95% CI)	-2.56 (-5.43, 0.31)	-4.51 (-6.89, -2.14)	-4.95 (-7.77, -2.13)
P	Ref.	0.30	0.30

Abbreviations: CI, confidence interval. FSMC, Fatigue Scale for Motor and Cognitive Functions.

[1] Adjusted means are least square means (LSM) and 95% CI from multivariable linear regression models. *P* values are from the pairwise comparison between the tertiles with tertile 1 as the reference group. Models were adjusted for treatment group of the original PUSH study, sex, reason for admission to rehabilitation, duration of rehabilitation, and baseline covariates: age, BMI, Charlson Comorbidity Index, vitamin D status, use of walking aids, magnesium tertiles, and measure of the outcome.

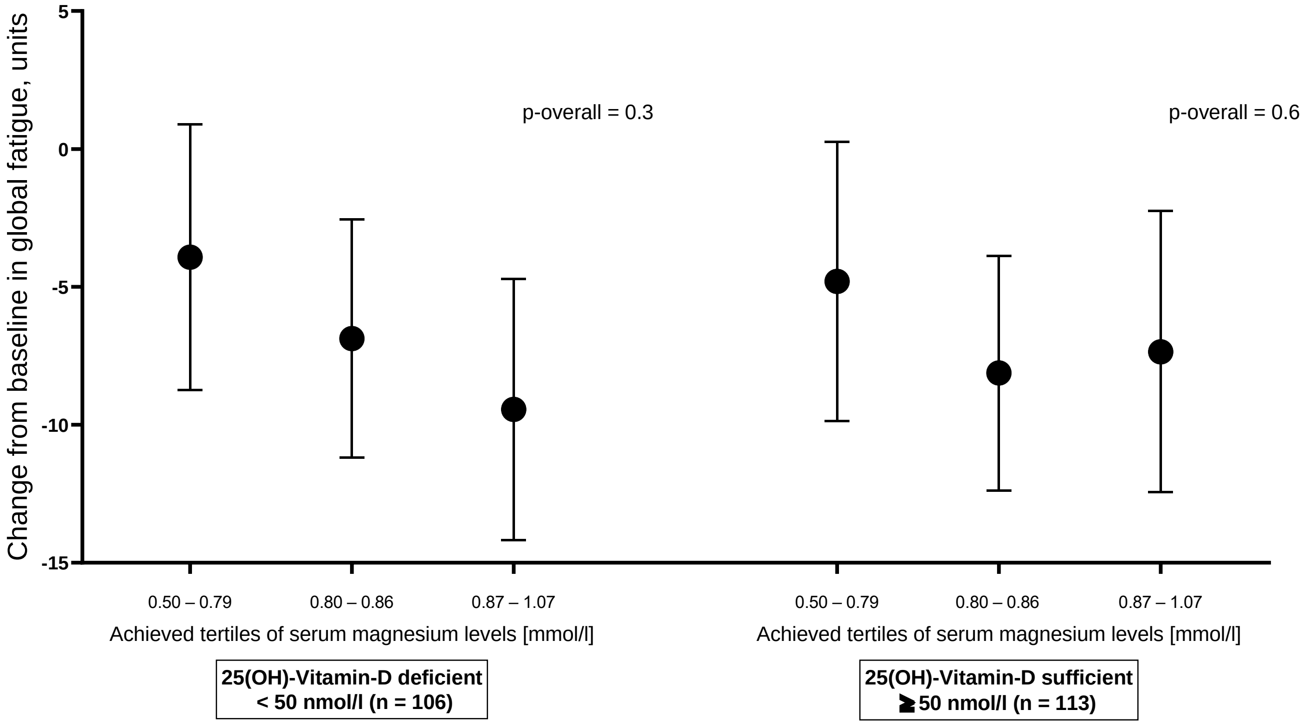

Supplemental Fig. 3 Adjusted[1] mean change in global fatigue (95% CI) after four weeks of rehabilitation by achieved tertiles of magnesium according to vitamin D status at week 4

[1] Adjusted means are least square means (LSM) and 95% CI from multivariable linear regression models. *P* values are from the pairwise comparison between the tertiles with tertile 1 as the reference group. Models were adjusted for treatment group of the original PUSH study, sex, reason for admission to rehabilitation, duration of rehabilitation, and baseline covariates: age, BMI, Charlson Comorbidity Index, vitamin D status, use of walking aids, magnesium tertiles, and measure of the outcome.

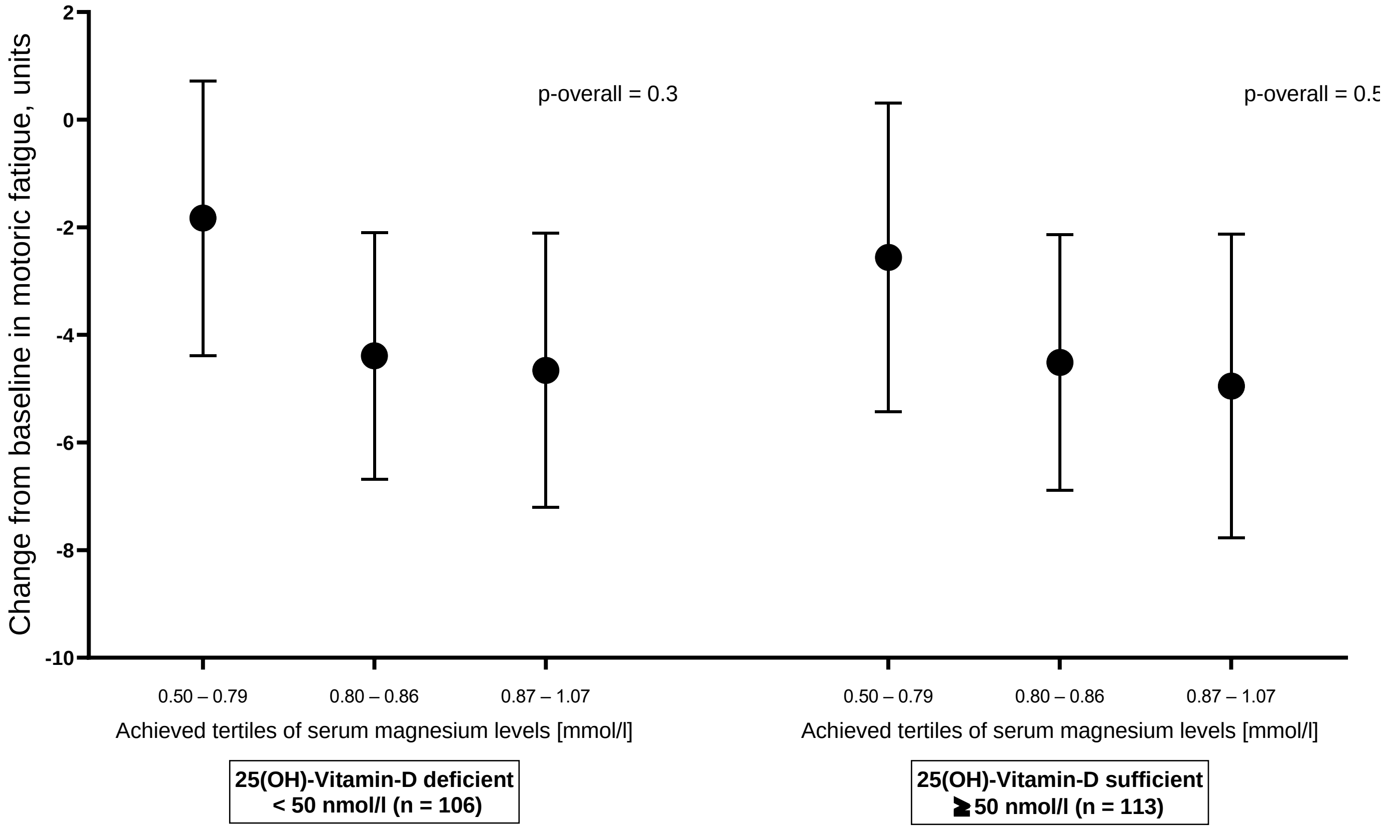

Supplemental Fig. 4 Adjusted[1] mean change in motor fatigue (95% CI) after four weeks of rehabilitation by achieved tertiles of magnesium according to vitamin D status at week 4

[1] Adjusted means are least square means (LSM) and 95% CI from multivariable linear regression models. *P* values are from the pairwise comparison between the tertiles with tertile 1 as the reference group. Models were adjusted for treatment group of the original PUSH study, sex, reason for admission to rehabilitation, duration of rehabilitation, and baseline covariates: age, BMI, Charlson Comorbidity Index, vitamin D deficiency (deficiency defined as 25(OH)D <50 nmol/l), use of walking aids, magnesium levels, and measure of the outcome.
